# Supplementary material for: Genome-Wide Computational Prediction and Analysis of Noncoding RNAs in Oleidesulfovibrio alaskensis G20
Source: Microorganisms. 2024 May 10;12(5):960. doi: 10.3390/microorganisms12050960 (PMC11124144; doi:10.3390/microorganisms12050960)

Supplemental figures of ncRNA structures identified in the genome of OA G20.

Supplemental_ Figure_S1: TPP (2 identified structures A and B and C: Rfam reference structure) and Cobalamin (2 identified structures D and E and F: Rfam reference structure) Riboswitches.

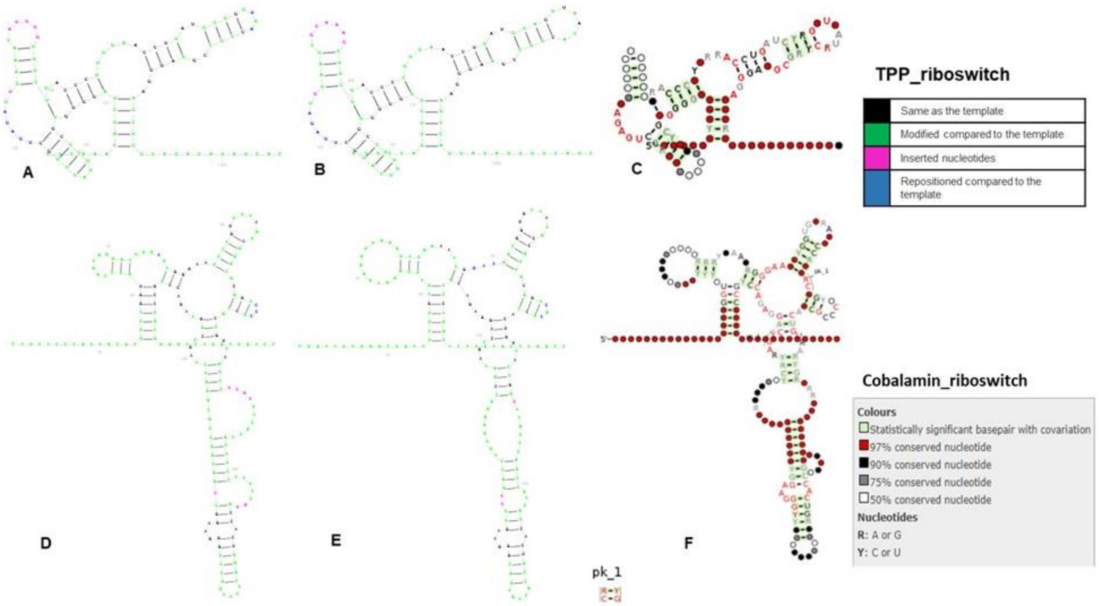

Supplement: Supplementary file 1 [file microorganisms-12-00960-s001.zip › Supplemental Figure S1.pdf]
