# Supplementary material for: Genome-Wide Computational Prediction and Analysis of Noncoding RNAs in Oleidesulfovibrio alaskensis G20
Source: Microorganisms. 2024 May 10;12(5):960. doi: 10.3390/microorganisms12050960 (PMC11124144; doi:10.3390/microorganisms12050960)
Supplement: Supplementary file 1 [file microorganisms-12-00960-s001.zip › Supplemental Figure S4.pdf]

Supplemental figures of ncRNA structures identified in the genome of OA G20.

Supplemental_Figure_S4: sX4 sRNAs (A-G: OA G20, H: Rfam reference structure)

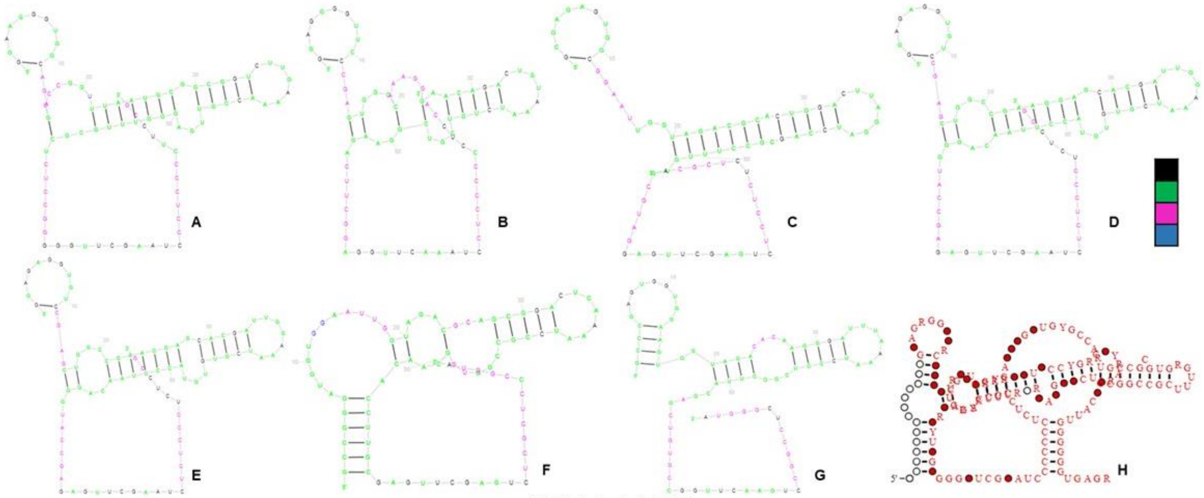

sX4: Proteobacterial sRNA
