# Supplementary material for: Genome-Wide Computational Prediction and Analysis of Noncoding RNAs in Oleidesulfovibrio alaskensis G20
Source: Microorganisms. 2024 May 10;12(5):960. doi: 10.3390/microorganisms12050960 (PMC11124144; doi:10.3390/microorganisms12050960)

Supplemental figures of ncRNA structures identified in the genome of OA G20.

Supplemental_Figure_S5: RNase P type (A: OA G20, B: Rfam reference structure), Hammerhead type II (C: OA G20, D: Rfam reference structure), tmRNA (E: OA G20, F: Rfam reference structure)

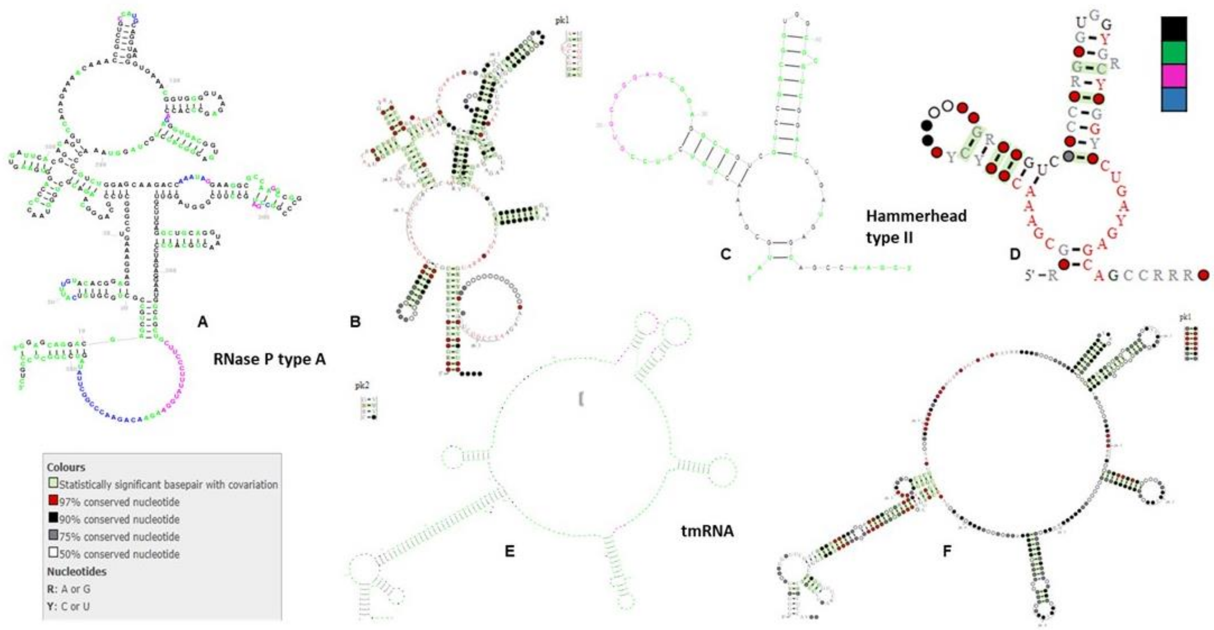

Supplement: Supplementary file 1 [file microorganisms-12-00960-s001.zip › Supplemental Figure S5.pdf]
