# Supplementary material for: Genome-Wide Computational Prediction and Analysis of Noncoding RNAs in Oleidesulfovibrio alaskensis G20
Source: Microorganisms. 2024 May 10;12(5):960. doi: 10.3390/microorganisms12050960 (PMC11124144; doi:10.3390/microorganisms12050960)
Supplement: Supplementary file 1 [file microorganisms-12-00960-s001.zip › Supplemental Table S2.pdf]

Supplemental Table S2: ncRNAs identified in OA G20 genome using approach 3

ncRNAs Family	Rfam ID	Gene type	Length	Genome location	Strand	Description
5S_rRNA	RF00001	rrf	115	3533731 : 3533617	-	5S ribosomal RNA
5S_rRNA	RF00001	rrf	115	2604159 : 2604045	-	5S ribosomal RNA
5S_rRNA	RF00001	rrf	115	1314473 : 1314587	+	5S ribosomal RNA
5S_rRNA	RF00001	rrf	115	74882 : 74996	+	5S ribosomal RNA
Glycine_riboswitch	RF00504	Cis-reg; riboswitch	110	2838359 : 2838468	+	Glycine riboswitch
TPP_riboswitch	RF00059	Cis-reg; riboswitch	104	1,573,919 : 1,574,022	+	TPP riboswitch (THI element)
TPP_riboswitch	RF00059	Cis-reg; riboswitch	107	1,574,080 : 1,574,186	+	TPP riboswitch (THI element)
FMN_riboswitch	RF00050	Cis-reg; riboswitch	164	1,078,058 : 1,078,221	+	FMN riboswitch
c-di-GMP-I	RF01051	Cis-reg; riboswitch	70	841,128 : 841,197	+	Cyclic di-GMP-I riboswitch
Cobalamin_riboswitch	RF00174	Cis-reg; riboswitch	183	3,093,545 : 3,093,727	+	Cobalamin riboswitch
Cobalamin_riboswitch	RF00174	Cis-reg; riboswitch	180	2,347,981 : 2,347,802	-	Cobalamin riboswitch
SAM-riboswitch class I	RF00162	Cis-reg; riboswitch	108	2,490,976 : 2,491,083	+	SAM riboswitch (S box leader)
Bacterial small_SRP	RF00169	ffs	96	1,028,491 : 1,028,396	-	Small signal recognition particle RNA (SRP)
6S	RF000013	SsrS_RNA	179	1,007,678 : 1,007,500	-	6S RNA
SSU_rRNA_bacteria	RF0177	rRNA	1542	2,609,189 : 2,607,648	-	Bacterial small subunit ribosomal RNA
SSU_rRNA_bacteria	RF0177	rRNA	1542	1,309,443 : 1,310,984	+	Bacterial small subunit ribosomal RNA
SSU_rRNA_bacteria	RF0177	rRNA	1542	3,538,761 : 3,537,220	-	Bacterial small subunit ribosomal RNA
SSU_rRNA_bacteria	RF0177	rRNA	1542	69,852 : 71,393	+	Bacterial small subunit ribosomal RNA
LSU_rRNA_bacteria	RF02541	rRNA	2931	2,607,209 : 2,604,279	-	Bacterial large subunit ribosomal RNA
LSU_rRNA_bacteria	RF02541	rRNA	2931	3,536,781 : 3,533,851	-	Bacterial large subunit ribosomal RNA
LSU_rRNA_bacteria	RF02541	rRNA	2932	71,832 : 74,762	+	Bacterial large subunit ribosomal RNA
LSU_rRNA_bacteria	RF02541	rRNA	2932	1,311,423 : 1,314,353	+	Bacterial large subunit ribosomal RNA
tRNA_Sec	RF01852	tRNA	90	1,531,711 : 1,531,804	-	tRNA-Sec
tRNA	RF00005	tRNA	73	2,047,102 : 2,047,175	+	tRNA
rnpB	RF00010	rnpB	361	1,785,161 : 1,785,521	-	Bacterial Rnase P class A
hammerhead	RF02276	DDE_RS18215	76	3,331,688 : 3,331,763	+	Hammerhead ribozyme (type II)
tmRNA	RF00023	SsrA	360	1,988,131 : 1,988,490	-	tmRNA
